# Three vs. Four Cycles of Neoadjuvant Chemotherapy for Localized Muscle Invasive Bladder Cancer Undergoing Radical Cystectomy: A Retrospective Multi-Institutional Analysis

**DOI:** 10.3389/fonc.2021.651745

**Published:** 2021-05-11

**Authors:** Matteo Ferro, Ottavio de Cobelli, Gennaro Musi, Giuseppe Lucarelli, Daniela Terracciano, Daniela Pacella, Tommaso Muto, Angelo Porreca, Gian Maria Busetto, Francesco Del Giudice, Francesco Soria, Paolo Gontero, Francesco Cantiello, Rocco Damiano, Fabio Crocerossa, Abdal Rahman Abu Farhan, Riccardo Autorino, Mihai Dorin Vartolomei, Matteo Muto, Michele Marchioni, Andrea Mari, Luca Scafuri, Andrea Minervini, Nicola Longo, Francesco Chiancone, Sisto Perdona, Pietro De Placido, Antonio Verde, Michele Catellani, Stefano Luzzago, Francesco Alessandro Mistretta, Pasquale Ditonno, Vincenzo Francesco Caputo, Michele Battaglia, Stefania Zamboni, Alessandro Antonelli, Francesco Greco, Giorgio Ivan Russo, Rodolfo Hurle, Nicolae Crisan, Matteo Manfredi, Francesco Porpiglia, Giuseppe Di Lorenzo, Felice Crocetto, Carlo Buonerba

**Affiliations:** ^1^ Division of Urology of European Institute of Oncology (IEO), IRCCS, Milan, Italy; ^2^ Department of Oncology and Hematology Oncology, Faculty of Medicine and Surgery, University of Milan, Milan, Italy; ^3^ Department of Emergency and Organ Transplantation, School of Medicine, University of Bari Aldo Moro, Bari, Italy; ^4^ Department of Translational Medical Sciences, University of Naples Federico II, Naples, Italy; ^5^ Department of Public Health, University of Naples Federico II, Naples, Italy; ^6^ Oncological Urology, Veneto Institute of Oncology IOV - IRCCS, Padua, Italy; ^7^ Department of Urology and Renal Transplantation, University of Foggia Policlinico Riuniti of Foggia, Foggia, Italy; ^8^ Department of Urology, Sapienza University of Rome, Rome, Italy; ^9^ Division of Urology, Department of Surgical Sciences, AOU Cittá della Salute e della Scienza, Torino School of Medicine, Turin, Italy; ^10^ Department of Urology, University of Catanzaro, UNIVERSITÁ “MAGNA GRÆCIA” di Catanzaro, Catanzaro, Italy; ^11^ Division of Urology, Virginia Commonwealth University Health System, Richmond, VA, United States; ^12^ Department of Urology, Vienna General Hospital, Vienna, Austria; ^13^ Department of Cell and Molecular Biology, George Emil Palade University of Medicine, Pharmacy, Sciences and Technology of Târgu Mureş, Tirgu Mures, Romania; ^14^ Department of Hematology, Oncology and Radiotherapy Azienda ospedaliera San Giuseppe Moscati, Avellino, Avelino, Italy; ^15^ Department of Urology, G. D’Annunzio University of Chieti-Pescara, Chieti, Italy; ^16^ Department of Experimental and Clinical Medicine, Unit of Oncologic Minimally-Invasive Urology and Andrology, University of Florence, Careggi University Hospital, Florence, Italy; ^17^ Department of Clinical Medicine and Surgery, School of Medicine and Surgery, University of Naples Federico II, Naples, Italy; ^18^ Department of Neuroscience, Reproductive and Odontostomatological Sciences, University of Naples Federico II, Naples, Italy; ^19^ Division of Urology, Hospital Antonio Cardarelli, Naples, Italy; ^20^ Division of Urology, Istituto Nazionale Tumori Fondazione G. Pascale (IRCCS), Naples, Italy; ^21^ Department of Urology, Civil Hospital of Brescia, Brescia, Italy; ^22^ Department of Urology, University of Verona, Azienda Ospedaliera Universitaria Integrata Verona - Polo Chirurgico Confortini - Borgo Trento, Verona, Italy; ^23^ Department of Urology, Humanitas Gavazzeni, IRRCS, Bergamo, Italy; ^24^ Department of Urology, University of Catania, Catania, Italy; ^25^ Department of Urology, IRCCS Humanitas Research Hospital, Milan, Italy; ^26^ Department of Urology, Iuliu Hațieganu University of Medicine and Pharmacy, Ciuj Napoca, Romania; ^27^ Urology Unit - Department of Oncology, School of Medicine, University of Turin, Turin, Italy; ^28^ Department of Urology, Humanitas Research Hospital Milano, Milan, Italy; ^29^ Department of Medicine and Health Science, University of Molise, Campobasso, Italy; ^30^ Rare Tumor Reference Center, Federico II University Hospital, Naples, Italy; ^31^ Centro di Referenza Nazionale per l’Analisi e Studio di Correlazione tra Ambiente, Animale e Uomo, Istituto Zooprofilattico Sperimentale del Mezzogiorno, Portici, Italy

**Keywords:** bladder cancer, neoadjuvant chemotherapy, radical cystectomy, observational study, cisplatin-based chemotherapy

## Abstract

**Background:**

Three or four cycles of cisplatin-based chemotherapy is the standard neoadjuvant treatment prior to cystectomy in patients with muscle-invasive bladder cancer. Although NCCN guidelines recommend 4 cycles of cisplatin-gemcitabine, three cycles are also commonly administered in clinical practice. In this multicenter retrospective study, we assessed a large and homogenous cohort of patients with urothelial bladder cancer (UBC) treated with three or four cycles of neoadjuvant cisplatin-gemcitabine followed by radical cystectomy, in order to explore whether three vs. four cycles were associated with different outcomes.

**Methods:**

Patients with histologically confirmed muscle-invasive UBC included in this retrospective study had to be treated with either 3 (cohort A) or 4 (cohort B) cycles of cisplatin-gemcitabine as neoadjuvant therapy before undergoing radical cystectomy with lymphadenectomy. Outcomes including pathologic downstaging to non-muscle invasive disease, pathologic complete response (defined as absence of disease -ypT0), overall- and cancer-specific- survival as well as time to recurrence were compared between cohorts A vs. B.

**Results:**

A total of 219 patients treated at 14 different high-volume Institutions were included in this retrospective study. Patients who received 3 (cohort A) vs. 4 (cohort B) cycles of neoadjuvant cisplatin-gemcitabine were 160 (73,1%) vs. 59 (26,9%).At univariate analysis, the number of neoadjuvant cycles was not associated with either pathologic complete response, pathologic downstaging, time to recurrence, cancer specific, and overall survival. Of note, patients in cohort B vs. A showed a worse non-cancer specific overall survival at univariate analysis (HR= 2.53; 95 CI= 1.05 - 6.10; p=0.046), although this finding was not confirmed at multivariate analysis.

**Conclusions:**

Our findings suggest that 3 cycles of cisplatin-gemcitabine may be equally effective, with less long-term toxicity, compared to 4 cycles in the neoadjuvant setting.

## Introduction

Bladder cancer represents approximately 3% of cancer diagnoses in the world, with a 4-time higher prevalence in males vs. females and >90% of cases diagnosed in individuals older than 55 years of age (1). Age-standardized incidence rates of approximately 20 cases per 100,000 per year in males and  4.5 cases per 100,000 per year in females are reported in Europe and North America (2), with certain areas of Italy showing remarkably higher rates [e.g. in the province of Naples, Italy, an age-standardized incidence rate of 75.3 and 16.3 cases per 100,0000 per year in males and females, respectively, has been reported (3)].

While chemotherapy agents such as mitomycin and gemcitabine (4) can be used for intravesical therapy against non-invasive bladder cancer, intravesical BCG represents the standard therapy for patients with T1 disease (5), and cystectomy (6–8), with or without perioperative chemotherapy (9), is recommended for muscle-invasive, localized bladder cancer (10). Advances in the field of peri-operative systemic therapy of muscle-invasive bladder cancer have been scarce over the past 20 years. Neoadjuvant chemotherapy regimens based on methotrexate, vinblastine, doxorubicin cisplatin or cisplatin-gemcitabine were associated with an absolute increase in 5-year survival of 8% in patients with muscle invasive-bladder cancer (9). Although neoadjuvant cisplatin-based chemotherapy is currently recommended by NCCN (10) and EAU (11) guidelines in patients with muscle-invasive UBC, the optimal chemotherapy schedule and number of cycles remain to be established. According to NCCN guidelines, dose-dense methotrexate, vinblastine, doxorubicin, cisplatin combination can be administered for 3 or 4 cycles, while cisplatin, methotrexate and vinblastine combination is administered for 3 cycles. Four cycles of gemcitabine and cisplatin represent a viable option in the perioperative setting, on the grounds of the results obtained in a large randomized phase III trial (12)and of retrospective case series (13). Nevertheless, the optimal number of cycles of cisplatin-gemcitabine remains therefore to be determined, given the lack of comparative studies specifically designed to assess optimal number of cycles.

In this multicenter retrospective study, we assessed a large and homogenous cohort of patients with histologically confirmed urothelial bladder cancer (UBC) treated with 3 vs. 4 neoadjuvant cisplatin-based chemotherapy followed by radical cystectomy, to explore potential differences in outcomes in terms of cancer specific-, non-cancer specific, overall- survival, time to recurrence and pathologic response and downstaging rates.

## Patients and Methods

### Patients

All patients with UBC treated with neoadjuvant chemotherapy at the participating Institutions from January 2000 until January 2015 had to be assessed for inclusion in this retrospective study. Patients were included in this retrospective study if they had histologically confirmed muscle-invasive bladder cancer with predominant urothelial component and were treated with 3 or 4 cycles of cisplatin-gemcitabine (gemcitabine 1000-1250 mg/m2 on days 1 and 8, and cisplatin 70 mg/m2 on day 1, every 3 weeks) as neoadjuvant therapy before undergoing radical cystectomy with lymphadenectomy. Only patients with cT1-4N0M0 on whole body CT scan with and without contrast prior to chemotherapy start and with a follow-up after surgery longer than 36 months were included in this retrospective study. Follow-up was conducted with a whole CT scan with and without contrast every 3-6 months and additional tests (MRI, bone scan) if clinically indicated. Data about sex, age, ECOG performance status, Charlson Comorbidity index, previous number of cycles of cisplatin-based neoadjuvant therapy, hemoglobin, creatinine, absolute neutrophil and lymphocyte counts, total and HDL cholesterol, CRP, presence of histologically proven positive lymph-nodes, and cancer-specific survival (months) were required to have been measured within 14 days and be fully available for the patient to be included in this retrospective study. Date and cause of death were collected using death certificates and ISTAT (Italian National Institute of Statistics) cause of death records. These variables were considered as potentially prognostic and assessed for their association with cancer specific survival. Information regarding recurrence was extracted from medical charts. Retrospective observation started on the day of cystectomy until death or last follow-up.

### Statistical Analysis

**Figure 1:**
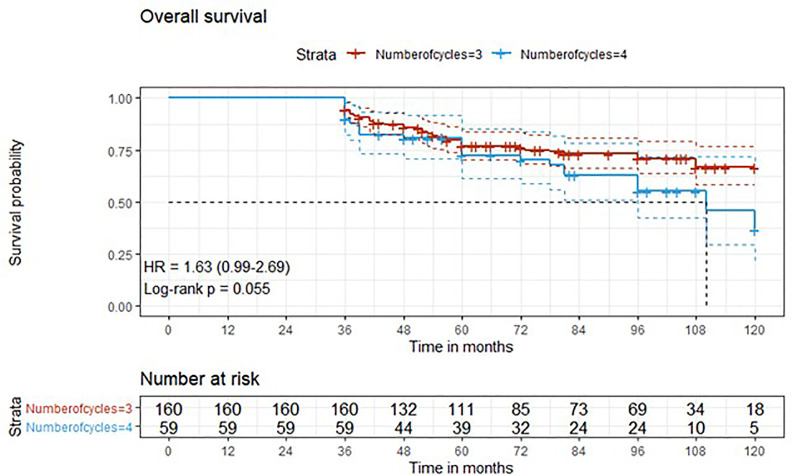
Kaplan-Meier curves with 95% CI (dashed lines). P-value computed with log-rank test (overall survival).

**Figure 2:**
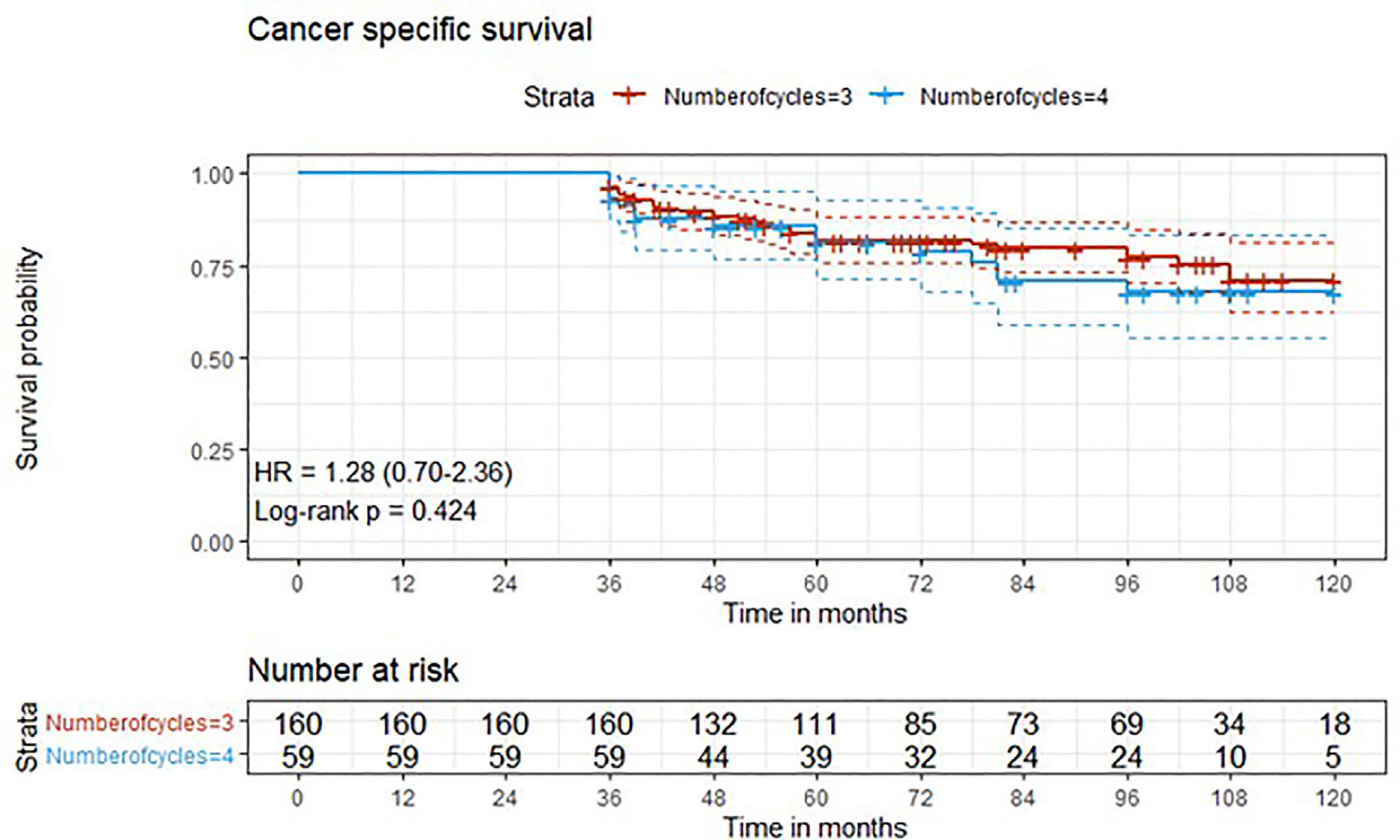
Kaplan-Meier curves with 95% CI (dashed lines). P-value computed with log-rank test (cancer specific survival).

**Figure 3:**
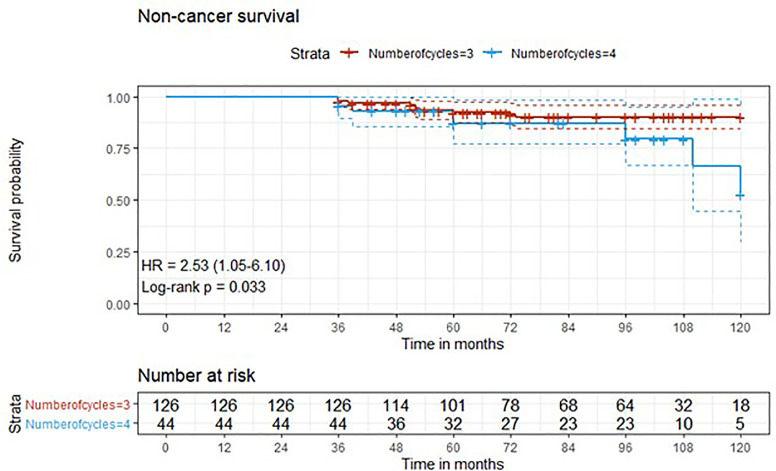
Kaplan-Meier curves with 95% CI (dashed lines). P-value computed with log-rank test (non-cancer survival).

**Figure 4:**
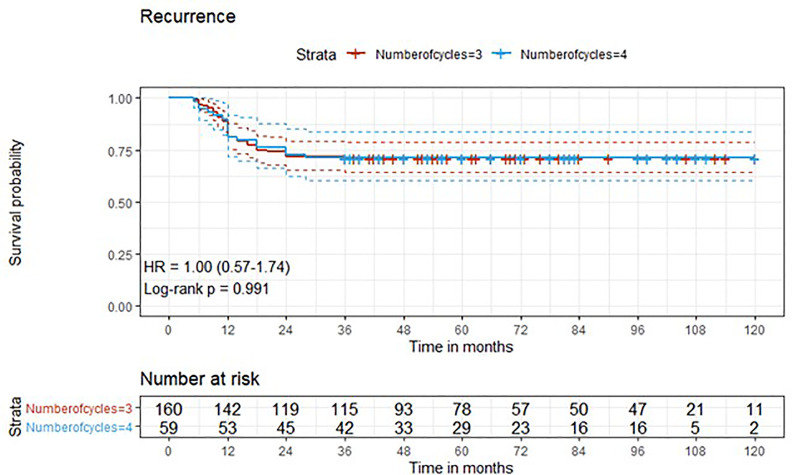
Kaplan-Meier curves with 95% CI (dashed lines). P-value computed with log-rank test (recurrence).

Descriptive statistics were used to describe the overall cohort with respect to the main demographical and clinical characteristics. Frequencies (percentages) were used for categorical variables, while medians (Q1; Q3), were used for quantitative variables. Associations between the number of cycles along with other potential predictive factors and the outcome variables downstaging and complete response were evaluated using univariate logistic regression models. Additionally, variables that presented a significant association in the univariate analysis were added to a multivariate logistic regression model. Time-to-event outcome variables were analyzed estimating survival curves with the Kaplan-Meier method and the difference between the curves was computed using log-rank test (**Figures 1**–**4**). The association of the number of cycles and other variables of potential interest with overall survival, cancer-specific survival and recurrence was conducted using univariate Cox regression. Factors that presented a p-value < 0.1 were added, along with the variable of interest (number of cycles), to a multivariate Cox regression model. The proportional hazard assumption was tested using the Schoenfeld residuals. For all analyses, a p-value < 0.05 was considered statistically significant. Analyses were performed using the statistical software R, version 4.0.3.

## Results

### Patients’ Baseline Characteristics

A total of 245 patients receiving neoadjuvant cisplatin-gemcitabine at 14 different high-volume Institutions were initially evaluated for inclusion in this retrospective study. After excluding 7 patients who received 6 cycles, 3 patients who received 1 cycle, 13 patients who received 2 cycles and 3 patients with missing data, a total of 219 patients receiving 3 or 4 cycles were included in this retrospective study. Patients who received 3 (cohort A) vs. 4 (cohort B) cycles of neoadjuvant cisplatin-gemcitabine were 160 (73,1%) vs. 59 (26,9%). In cohort A, 83.1% were males and median age was 66 (IQR = 59,000 to 72,00).

In cohort B, median age was 66 years (IQR = 59,000 to 72,00), and 81.4% were males. Patients’ characteristics are detailed in **Table 1**.

**Table 1:** Characteristics of the study population.

	Number of cycles of cisplatin-gemcitabine				p-value
	3 (COHORT A)		4 (COHORT B)		
	Median (IQR range)	Mean (SD)	Median (IQR range)	Mean (SD)	
Age	66 (59, 72)	66 (10)	66 (60, 74)	67 (10)	0.762
Basophils x10^3^/µL	0.03 (0.02, 0.05)	0.05 (0.06)	0.03 (0.01, 0.05)	0.04 (0.05)	0.283
Charlson index	3.00 (2.00, 5.00)	3.79 (2.11)	2.00 (2.00, 3.00)	2.73 (1.48)	**<0.001**
Eosinophils x10^3^/µL	0.12 (0.08, 0.22)	0.20 (0.27)	0.13 (0.07, 0.21)	0.28 (0.83)	0.817
Lymphocytes x10^3^/µL	1.88 (1.50, 2.41)	2.42 (2.91)	1.81 (1.43, 2.41)	2.17 (2.14)	0.344
Monocytes x10^3^/µL	0.70 (0.45, 1.19)	1.25 (1.48)	0.72 (0.54, 1.04)	1.14 (1.17)	0.427
Neutrophils x10^3^/µL	4.39 (3.20, 5.74)	6.10 (9.05)	4.44 (3.42, 5.89)	5.87 (9.06)	0.725
NLR	2.21 (1.59, 3.25)	2.71 (1.66)	2.30 (1.70, 3.75)	2.87 (1.67)	0.553
PCR (mg/L)	8 (4, 13)	10 (10)	8 (5, 13)	10 (8)	0.929
Platelets x10^3^/µL	232 (190, 320)	261 (104)	253 (187, 334)	260 (87)	0.750
Preoperative PCR (mg/L)	7 (3, 12)	9 (7)	5 (2, 8)	6 (6)	**0.033**
Albumin (g/dl)	3.90 (3.50, 4.21)	3.84 (0.53)	3.80 (3.40, 4.05)	3.69 (0.49)	0.065
BMI	26.0 (23.0, 28.0)	25.9 (3.6)	25.1 (23.2, 28.6)	25.9 (3.7)	0.903
Creatinine (mg/dl)	1.05 (0.87, 1.38)	1.22 (0.70)	0.97 (0.85, 1.23)	1.08 (0.37)	0.145
Fibrinogen level (mg/dl)	2.58 (2.09, 4.25)	3.24 (1.27)	2.23 (2.04, 3.59)	2.89 (1.07)	0.119
HB (g/dl)	12.85 (11.0, 14.4)	15.34 (17.43)	12.5 (11.25, 14.4)	12.72 (1.94)	0.938
HDL cholesterol (mg/dl)	45 (36, 57)	48 (14)	55 (44, 64)	52 (14)	**0.035**
Total cholesterol level (mg/dl)	198 (175, 210)	201 (37)	203 (184, 230)	206 (40)	0.225
SED rate (mm/h)	19 (8, 30)	21 (16)	15 (6, 23)	17 (13)	0.204
	**Absolute Number**	**%**	**Absolute Number**	**%**	
Gender					
Males	133	83.1%	48	81.4%	0.916
Females	27	16.9%	11	18.6%	
ECOG Performance status					
0	79	49.4%	44	74.6%	**0.007**
1	62	38.7%	9	15.2%	
2	10	6.3%	3	5.1%	
3	4	2.5%	1	1.7%	
4	5	3.1%	2	3.4%	
Clinical T stage					0.65
<=2	95	59.7%	37	62.7%	
>=3	65	40.3%	22	37.3	
Clinical N stage					
0	160	100%	59	100%	
Pathologic stage					
Ta	6	3.7%	3	5.1%	0.110
T1	11	6.9%	7	11.9%	
T2	40	25.0%	6	10.2%	
T3	43	26.9%	16	27.1%	
T4	14	8.7%	11	18.6%	
T0	36	22.5%	13	22.0%	
Tis	10	6.3%	3	5.1%	
N0	110	68.8%	39	66.1%	0.073
N1	19	11.9%	4	6.8%	
N2	18	11.2%	11	18.6%	
N3	5	3.1%	5	8.5%	
Nx	8	5.0%	0	0.0%	

P-values computed with Student’s t test or Kruskal Wallis test as appropriate for continuous variables, and with Chi-square test or Fisher’s exact test as appropriate for categorical variables. Significant values are highlighted in bold. BMI, Body Mass Index; SED rate, Sedimentation rate.

### Outcomes

After a median follow-up of 76 months, median overall survival was not reached in cohort A, with 43 reported deaths (34 because of bladder cancer), while it was 110 months (95%CI: 81-120) in cohort B, with 24 reported deaths (15 because of bladder cancer). Median cancer-specific survival and time to recurrence were not reached in either cohort. Pathologic downstaging to non-muscle invasive disease was reported in 129 and 43 patients in cohort A and B, respectively. Complete pathologic response was reported in 36 (22.5%) and 13 (22.0%) patients in cohort A and B, respectively, while recurrence was reported in 46 (28.7%) vs. 17 (28.8%) patients in cohorts A vs. B, respectively.

Three-year estimated OS probability was 89.8% (82.4-97.9) for cohort A compared to 94.4% (90.9-98.0) for cohort B. Three-year estimated cancer-specific survival probability was 96.3% (93.4-99.2) for cohort A compared to 93.2% (87.9-99.9) for cohort B. Three-year estimated OS probability was 92.4% (88.4-96.4) for those who did not have a complete response compared to 95.9% (90.5-100.0) for those who had a complete response. Three-year estimated recurrence-free survival probability was 71.3% (64.6-78.6) for cohort A compared to 71.2% (60.5-83.7) for cohort B. Three-year estimated non-cancer survival probability was 97.6% (95.0-100.0) for cohort A compared to 95.5% (89.5-100.0) for cohort B. Three-year estimated cancer-specific survival probability was 94.7% (91.4-98.1) for those who did not have a complete response compared to 98.0% (94.1-100.0) for those who had a complete response. Three-year estimated OS probability was 87.2% (78.2-97.3) for those who did not have downstaging compared to 94.8% (91.5-98.2) for those who had downstaging. Three-year estimated cancer-specific survival probability was 89.4% (81.0-98.6) for those who did not have downstaging compared to 97.1% (94.6-99.6) for those who had downstaging. Three-year estimated recurrence-free survival probability was 68.1% (56.0-82.8) for those who did not have downstaging compared to 72.1% (65.7-79.1) for those who had downstaging.

Five-year estimated OS probability was 76.9% (70.4-83.9) for cohort A compared to 72.3% (61.3-85.4) for cohort B. Five-year estimated cancer-specific survival probability was 81.7% (75.6-88.2) for cohort A compared to 81.1% (71.2-92.5) for cohort B. Five-year estimated recurrence-free survival probability was 71.3% (64.6-78.6) for cohort A compared to 71.2% (60.5-83.7) for cohort B. Five-year estimated non-cancer survival probability was 92.4% (87.7-97.3) for cohort A compared to 87.1% (77.1-98.4) for cohort B. Five-year estimated OS probability was 72.1% (65.5-79.5) for those who did not have a complete response compared to 88.4% (79.2-98.6) for those who had a complete response. Five-year estimated cancer-specific survival probability was 77.7% (71.5-84.6) for those who did not have a complete response compared to 95.2% (88.9-100.0) for those who had a complete response. Five-year estimated recurrence-free survival probability was 71.8% (65.3-78.9) for those who did not have a complete response compared to 69.4% (57.6-83.6) for those who had a complete response. Five-year estimated OS probability was 69.7% (57.6-84.3) for those who did not have downstaging compared to 77.2% (70.1-84.1) for those who had downstaging. Five-year estimated cancer-specific survival probability was 73.6% (61.8-87.7) for those who did not have downstaging compared to 83.6% (77.8-89.7) for those who had downstaging. Five-year estimated recurrence-free survival probability was 68.1% (56.0-82.8) for those who did not have downstaging compared to 72.1% (65.7-79.1) for those who had downstaging.

### Univariate and Multivariate Analysis

At univariate analysis (**Tables 1**–**7**), the number of neoadjuvant cycles was not associated with either pathologic complete response, pathologic downstaging, time to recurrence, cancer specific, and overall survival. Of note, patients in cohort B vs. A showed a worse non-cancer specific overall survival at univariate analysis (HR= 2.53; 95 CI= 1.05- 6.10; p=0.046), although this finding was not confirmed at multivariate analysis.

**Table 2:** Uni- and multi-variate analysis of the number of cycles and other baseline variables as potential predictors of downstaging.

Variable	Downstaging					
	Univariate			Multivariate		
	OR	95% CI	p-value	aOR	95% CI	p-value
Number of neoadjuvant cisplatin-gemcitabine cycles	0.65	0.33, 1.32	0.224	–	–	–
Clinical T stage						
<=2	Reference	–	Reference			
>=3	0.86	0.45, 1.67	0.655	–	–	–
Sex						
** Male**	Reference	–	Reference			
** Female**	1.56	0.65, 4.37	0.352	–	–	–
Age	0.96	0.93, 0.99	**0.017**	0.99	0.95, 1.03	0.662
Ecog	0.57	0.41, 0.78	**<0.001**	0.70	0.44, 1.09	0.120
Charlson index	1.00	0.86, 1.19	0.960	–	–	–
HB (g/dl)	0.99	0.97, 1.01	0.148	–	–	–
Creatinine (mg/dl)	1.47	0.81, 3.36	0.246	–	–	–
PCR (mg/L)	0.95	0.91, 0.98	**0.004**	0.95	0.89, 1.01	0.131
VES (mm/h)	0.98	0.96, 1.00	**0.025**	0.99	0.97, 1.02	0.549
NLR	0.87	0.72, 1.04	0.130	–	–	–
Neutrophils x10^3/µL	0.99	0.96, 1.03	0.487	–	–	–
Lymphocytes x10^3/µL	0.99	0.89, 1.15	0.881	–	–	–
Monocytes x10^3/µL	1.06	0.84, 1.40	0.634	–	–	–
Eosinophils x10^3/µL	1.09	0.59, 3.38	0.812	–	–	–
Basophils x10^3/µL	0.36	0.00, 176	0.731	–	–	–
Platelets x10^3/µL	1.00	1.00, 1.00	0.962	–	–	–
Albumin (g/dl)	1.44	0.78, 2.66	0.246	–	–	–
BMI	1.10	1.00, 1.21	**0.048**	1.05	0.95, 1.16	0.347
Total cholesterol level (mg/dl)	0.99	0.99, 1.00	0.134	–	–	–
HDL (mg/dl)	0.99	0.97, 1.01	0.449	–	–	–
Fibrinogen level (mg/dl)	0.92	0.71, 1.19	0.507	–	–	–
Preoperative PCR (mg/L)	0.95	0.91, 0.99	**0.025**	1.01	0.94, 1.08	0.847

P-values and OR are computed with single and multiple logistic regression. Significant values are highlighted in bold.

**Table 3:** Uni- and multi-variate analysis of the number of cycles and other baseline variables as potential predictors of complete response.

Variable	Complete pathologic response					
	Univariate			Multivariate		
	OR	95% CI	p-value	aOR	95% CI	p-value
Number of cycles	0.97	0.46, 1.96	0.941	–	–	–
Clinical T stage						
** <=2**	Reference	–	Reference			
** >=3**	1.47	0.77, 2.78	0.243	–	–	–
Sex						
** Male**	Reference	–	Reference			
** Female**	0.75	0.29, 1.73	0.512	–	–	–
Age	0.98	0.95, 1.01	0.158	–	–	–
Ecog	0.81	0.53, 1.16	0.254	–	–	–
Charlson index	0.96	0.81, 1.13	0.648	–	–	–
HB (g/dl)	1.00	0.98, 1.02	0.647	–	–	–
Creatinine (mg/dl)	0.80	0.39, 1.36	0.460	–	–	–
PCR (mg/L)	1.00	0.96, 1.03	0.931	–	–	–
VES (mm/h)	0.98	0.95, 1.00	**0.043**	0.98	0.95, 1.00	**0.084**
NLR	0.93	0.75, 1.12	0.450	–	–	–
Neutrophils x10^3/µL	1.01	0.97, 1.04	0.519	–	–	–
Lymphocytes x10^3/µL	1.05	0.94, 1.17	0.364	–	–	–
Monocytes x10^3/µL	1.30	1.06, 1.62	**0.012**	1.28	1.04, 1.58	**0.019**
Eosinophils x10^3/µL	1.03	0.42, 1.87	0.932	–	–	–
Basophils x10^3/µL	0.19	0.00, 64.7	0.599	–	–	–
Platelets x10^3/µL	1.00	1.00, 1.00	0.499	–	–	–
Albumin (g/dl)	1.13	0.61, 2.10	0.703	–	–	–
BMI	1.05	0.96, 1.15	0.269	–	–	–
Total cholesterol level (mg/dl)	1.00	1.00, 1.01	0.281	–	–	–
HDL (mg/dl)	1.00	0.97, 1.02	0.709	–	–	–
Fibrinogen level (mg/dl)	0.91	0.69, 1.18	0.491	–	–	–
Preoperative PCR (mg/L)	0.99	0.94, 1.04	0.719	–	–	–

P-values and OR are computed with single and multiple logistic regression. Significant values are highlighted in bold.

**Table 4:** Uni- and multi-variate analysis of the number of cycles and other baseline variables as potential predictors of overall survival.

Variable	Overall survival					
	Univariate			Multivariate		
	HR	95% CI	p-value	aHR	95% CI	p-value
Number of cycles						
** 3**	Reference	–	Reference	Reference	–	Reference
** 4**	1.63	0.99, 2.69	0.061	1.66	0.99, 2.80	0.056
Clinical T stage						
** <=2**	Reference	–	Reference			
** >=3**	0.85	0.52, 1.41	0.532	–	–	–
Sex						
** Male**	Reference	–	Reference	–	–	–
** Female**	1.57	0.90, 2.76	0.129	–	–	–
Age	0.97	0.73, 1.28	0.811	–	–	–
Ecog	0.97	0.85, 1.11	0.644	–	–	–
Charlson index	1.00	0.98, 1.02	0.992	–	–	–
HB (g/dl)	0.65	0.37, 1.14	0.083	1.00	0.98, 1.02	0.968
Creatinine (mg/dl)	1.02	0.99, 1.04	0.250	–	–	–
PCR (mg/L)	1.02	1.00, 1.03	**0.013**	1.01	0.99, 1.03	0.374
VES (mm/h)	1.25	1.10, 1.42	**0.001**	1.01	1.00, 1.03	0.142
NLR	1.03	1.01, 1.05	**0.011**	1.16	1.01, 1.34	**0.040**
Neutrophils x10^3/µL	1.04	0.96, 1.12	0.397	–	–	–
Lymphocytes x10^3/µL	1.06	0.90, 1.25	0.514	–	–	–
Monocytes x10^3/µL	1.55	1.15, 2.07	**0.030**	1.12	0.96, 1.32	0.160
Eosinophils x10^3/µL	0.78	0.01, 70.9	0.915	–	–	–
Basophils x10^3/µL	1.00	1.00, 1.00	0.688	–	–	–
Platelets x10^3/µL	0.63	0.39, 1.00	0.050	1.00	1.00, 1.00	0.641
Albumin (g/dl)	0.91	0.85, 0.98	**0.007**	0.68	0.42, 1.12	0.131
BMI	1.01	1.01, 1.02	**<0.001**	0.95	0.88, 1.03	0.187
Total cholesterol level (mg/dl)	1.01	0.99, 1.02	0.401	–	–	–
HDL (mg/dl)	0.89	0.73, 1.10	0.276	–	–	–
Fibrinogen level (mg/dl)	1.00	0.97, 1.04	0.973	–	–	–
Preoperative PCR (mg/L)	1.00	0.97, 1.02	0.766	–	–	–

P-values and HR are computed with single and multiple Cox regression. Significant values are highlighted in bold.

**Table 5:** Uni- and multi-variate analysis of the number of cycles and other baseline variable as potential predictors of cancer-specific survival.

Variable	Cancer specific survival					
	Univariate			Multivariate		
	HR	95% CI	p-value	aHR	95% CI	p-value
Number of cycles						
** 3**	Reference	–	Reference	Reference	–	Reference
** 4**	1.28	0.70, 2.36	0.428	1.14	0.59, 2.20	0.690
Clinical T stage						
** <=2**	Reference	–	Reference			
** >=3**	0.81	0.45, 1.46	0.485	–	–	–
Sex						
** Male**	Reference	–	Reference			
** Female**	1.28	0.64, 2.56	0.499	–	–	–
Age	0.99	0.96, 1.02	0.628	–	–	–
Ecog	1.02	0.75, 1.40	0.888	–	–	–
Charlson index	1.05	0.92, 1.22	0.470	–	–	–
HB (g/dl)	1.00	0.98, 1.02	0.756	–	–	–
Creatinine (mg/dl)	0.44	0.20, 0.99	**0.018**	0.56	0.23, 1.34	0.194
PCR (mg/L)	1.02	0.99, 1.05	0.184	–	–	–
VES (mm/h)	1.03	1.01, 1.04	**0.002**	1.01	0.99, 1.03	0.339
NLR	1.34	1.17, 1.54	**<0.001**	1.07	0.89, 1.30	0.462
Neutrophils x10^3/µL	1.04	1.02, 1.05	**0.002**	1.02	0.99, 1.04	0209
Lymphocytes x10^3/µL	1.06	0.99, 1.13	0.179	–	–	–
Monocytes x10^3/µL	1.15	0.98, 1.35	0.129	–	–	–
Eosinophils x10^3/µL	1.66	1.24, 2.22	**0.014**	1.20	0.79, 1.84	0.397
Basophils x10^3/µL	1.23	0.01, 205	0.937	–	–	–
Platelets x10^3/µL	1.00	1.00, 1.00	0.181	–	–	–
Albumin (g/dl)	0.54	0.32, 0.94	**0.030**	0.64	0.34, 1.19	0.160
BMI	0.92	0.85, 1.00	**0.045**	0.94	0.85, 1.03	0.202
Total cholesterol level (mg/dl)	1.02	1.01, 1.03	**<0.001**	1.02	1.01, 1.02	**<0.001**
HDL (mg/dl)	1.00	0.98, 1.02	0.852	–	–	–
Fibrinogen level (mg/dl)	1.00	0.80, 1.26	0.972	–	–	–
Preoperative PCR (mg/L)	1.00	0.96, 1.04	0.993	–	–	–

P-values and HR are computed with single and multiple Cox regression. Significant values are highlighted in bold.

**Table 6:** Uni- and multi-variate analysis of the number of cycles and other baseline variable as potential predictors of non-cancer specific survival.

Variable	Non-cancer survival, N = 170					
	Univariate			Multivariate		
	HR	95% CI	p-value	aHR	95% CI	p-value
Number of cycles						
3	Reference	–	Reference	Reference	–	Reference
4	2.53	1.05, 6.10	**0.046**	1.91	0.77, 4.74	0.160
Clinical T stage						
** <=2**	Reference	–	Reference			
** >=3**	0.97	0.40, 2.38	0.947	–	–	–
Sex						
Male	Reference	–	Reference	–	–	–
Female	2.23	0.86, 5.79	0.123	–	–	–
Age at bc diagnosis	1.00	0.96, 1.05	0.933	–	–	–
Ecog	0.82	0.46, 1.47	0.484	–	–	–
Charlson index	0.76	0.57, 1.02	**0.050**	0.79	0.59, 1.07	0.127
HB (g/dl)	0.97	0.86, 1.10	0.481	–	–	–
Creatinine (mg/dl)	0.95	0.50, 1.80	0.877	–	–	–
PCR (mg/L)	1.00	0.95, 1.06	0.898	–	–	–
VES (mm/h)	1.00	0.97, 1.03	0.995	–	–	–
NLR	0.99	0.70, 1.41	0.974	–	–	–
Neutrophils x10^3/µL	0.85	0.66, 1.09	0.138	–	–	–
Lymphocytes x10^3/µL	0.60	0.32, 1.15	0.091	0.61	0.30, 1.22	0.163
Monocytes x10^3/µL	0.64	0.35, 1.14	0.069	0.71	0.37, 1.36	0.299
Eosinophils x10^3/µL	0.32	0.01, 9.37	0.458	–	–	–
Basophils x10^3/µL	0.39	0.00, 1,67	0.821	–	–	–
Platelets x10^3/µL	1.00	0.99, 1.00	0.528	–	–	–
Albumin (g/dl)	0.99	0.41, 2.35	0.975	–	–	–
BMI	0.87	0.76, 0.99	**0.035**	0.88	0.77, 1.01	0.724
Total cholesterol level (mg/dl)	0.99	0.98, 1.01	0.350	–	–	–
HDL (mg/dl)	1.01	0.98, 1.04	0.470	–	–	–
Fibrinogen level (mg/dl)	0.56	0.34, 0.92	**0.008**	0.50	0.29, 0.87	**0.014**
Preoperative PCR (mg/L)	1.00	0.94, 1.06	0.958	–	–	–

P-values and HR are computed with single and multiple Cox regression. Significant values are highlighted in bold.

**Table 7:** Uni- and multi-variate analysis of the number of cycles and other baseline variable as potential predictors of time to recurrence.

Variable	Time to Recurrence					
	Univariate			Multivariate		
	HR	95% CI	p-value	aHR	95% CI	p-value
Number of cycles						
** 3**	Reference	–	Reference	Reference	–	Reference
** 4**	1.00	0.57, 1.74	0.996	1.13	0.63, 2.01	0.681
Clinical T stage						
** <=2**	Reference	–	Reference			
** >=3**	1.11	0.68, 1.83	0.676	–	–	–
Sex						
** Male**	Reference	–	Reference	–	–	–
** Female**	1.12	0.60, 2.11	0.722	–	–	–
Age	1.01	0.98, 1.03	0.604	–	–	–
Ecog	1.26	0.99, 1.59	0.078	1.23	0.96, 1.57	0.095
Charlson index	1.01	0.89, 1.15	0.830	–	–	–
HB (g/dl)	0.97	0.91, 1.03	0.123	–	–	–
Creatinine (mg/dl)	0.89	0.57, 1.38	0.569	–	–	–
PCR (mg/L)	0.99	0.96, 1.02	0.506	–	–	–
VES (mm/h)	1.00	0.98, 1.02	0.924	–	–	–
NLR	1.04	0.90, 1.20	0.610	–	–	–
Neutrophils x10^3/µL	0.97	0.92, 1.03	0.184	–	–	–
Lymphocytes x10^3/µL	0.88	0.70, 1.11	0.131	–	–	–
Monocytes x10^3/µL	0.99	0.83, 1.18	0.909	–	–	–
Eosinophils x10^3/µL	0.66	0.24, 1.83	0.310	–	–	–
Basophils x10^3/µL	14.3	0.27, 761	0.215	–	–	–
Platelets	1.33	1.07, 1.66	**0.015**	1.31	1.05, 1.63	**0.016**
Albumin (g/dl)	1.00	0.63, 1.58	0.986	–	–	–
BMI	1.00	0.94, 1.07	0.982	–	–	–
Total cholesterol level (mg/dl)	1.00	1.00, 1.01	0.596	–	–	–
HDL (mg/dl)	0.99	0.97, 1.01	0.354	–	–	–
Fibrinogen level (mg/dl)	1.10	0.90, 1.33	0.353	–	–	–
Preoperative PCR (mg/L)	1.00	0.97, 1.04	0.933	–	–	–

P-values and HR are computed with single and multiple Cox regression. Significant values are highlighted in bold.

At multivariate analysis (**Tables 1** **–** **7**), Erythrocyte Sedimentation Rate was associated with complete pathologic response (p=0.084; HR=0,98; 95% CI: 0,95 to 1,00), NLR was associated with overall survival (p= 0,040; HR=1,16; 95% CI: 1,01 to 1,34), preoperative total cholesterol was associated with cancer-specific survival (p= 0,001; HR=1,02; 95% CI: 1,01 to 1,02), preoperative fibrinogen was associated with non-cancer specific survival (HR=0.50; 95% CI= 0.29 to 0.87; p=0.014), platelet count was associated with time to recurrence (p=0,016; HR=1,00; 95% CI: 1,00 to 1,00).

## Discussion

Neo-adjuvant chemotherapy based on cisplatin has represented the standard of care in patients with T2-T4 UBC for the past two decades (14), although the optimal schedule and number of cycles to administer remain to be determined (9). In a landmark meta-analysis (9) that analyzed data collected in 15 randomized trials enrolling a total of 3,285 patients neoadjuvant regimens based on cisplatin alone were not associated with any survival benefit, which was only provided by cisplatin-containing regimens including cisplatin-gemcitabine or MVAC ‐like chemotherapy (HR, 0.82; 95% CI, 0.74–0.91; p <.001; p = .99 for heterogeneity, I2 = 0%). This meta-analysis did not identify any differences in outcomes associated with GC vs. MVAC. Data collected from 12 trials of 1,734 patients, including 1,067 patients receiving gemcitabine-cisplatin and 667 patients receiving MVAC, showed that pCR was 25.7% in patients treated with CG and 24.3% in those receiving MVAC. Similarly, data collected from 10 trials of 1,495 patients, including 898 patients receiving GC and 597 patients receiving MVAC, showed no significant difference between GC and MVAC in terms of pathologic downstaging rate (odds ratio, 1.07; 95% CI, 0.85–1.34). Finally, data collected from 7 trials studies, including 1,414 patients, showed that GC vs. MVAC was associated with worse overall survival (HR, 1.26; 95% CI, 1.01–1.57; p = .94 for heterogeneity, I2 = 0%), although this difference was not statistically significant after excluding patients treated with carboplatin (9).

In a phase II trial that randomized 237 bladder cancer patients to dose dense MVAC, administered every 14 days for 4 cycles, or gemcitabine-cisplatin, administered every 21 days for 4 cycles, the pT0 rates for ddMVAC and GC were 32% and 35%, respectively (15). In a recently published randomized phase III trial (12) designed to compare the efficacy of dose dense (dd)-MVAC or GC in the neoadjuvant/adjuvant setting, 500 patients were randomized to either either six cycles of dd-MVAC every 2 weeks or four cycles of GC every 3 weeks. Of note, in the neoadjuvant group, 218 patients were treated with dd-MVAC (60% received the planned six cycles) and 219 were treated with GC (84% received the planned four cycles). A complete pathological response rate of 42% and 36% was obtained in the dd-MVAC vs. GC arms. In a retrospective observational study that included data from 212 patients muscle-invasive UBC treated with neoadjuvant chemotherapy (146 patients treated with GC and 66 patients treated with MVAC, the pCR rate was 29% in the MVAC cohort and 31% in the GC cohort, with a median of 3 cycles of chemotherapy administered and no significant difference in the pathologic response rate among the two regimens (16). In another retrospective study (13) including 42 patients receiving 4 cycles of neoadjuvant GC, the complete pathologic response rate was 26% (95% confidence interval [CI], 14-42), and no residual muscle-invasive disease proportion (<pT2) was 36% (95% CI, 21-52); pT0 was achieved in 28% (95% CI, 16-42) and <pT2 in 35% (95% CI, 23-49) of 54 MVAC-treated patients. All 15 GC patients achieving <pT2 pathologic stage remained disease-free at a median follow-up of 30 months.

In another retrospective study, including 58 patients treated with neoadjuvant GC therapy and 74 treated with neoadjuvant MVAC, similar pathologic complete response rates were obtained (20.7% vs. 18.9%, P = 0.83, respectively). Of note, while neoadjuvant GC yielded improved 2-year OS rate than neoadjuvant MVAC for clinical T2 disease (95.2% vs. 70.8%, P = 0.036), in patients with T3 or more advanced disease, neoadjuvant MVAC provided more pT0 (20.0% vs. 5.6%, P = 0.07) and better 2-year OS than neoadjuvant GC (71.1% vs. 55.0%, P = 0.142), although the difference was not statistically significant (17).

Of note, in our retrospective study, we obtained a complete pathologic response rate of 22,5% in patients receiving 3 cycles of neoadjuvant cisplatin-gemcitabine and of 22% in patients receiving 4 cycles of cisplatin-gemcitabine. These results are consistent with the published data reviewed. Pathologic complete response rates obtained with immune checkpoint inhibitors may be higher. While The PURE-01 trial reported that pembrolizumab was associated with a 37% complete response (pT0) after neoadjuvant therapy, the ABACUS trial showed that atezolizumab yielded a complete response rate of 31%. Conversely, preoperative combination of ipilimumab + nivolumab (18), cisplatin plus gemcitabine plus pembrolizumab (19), durvalumab + tremelimumab (20), was associated with a complete pathologic response rate of 46%, 44, 34.8%, respectively. Combination of 4 cycles of cisplatin-gemcitabine plus 4 cycles of nivolumab in the BLASST-1 trial was able to yield a complete pathologic response rate in 20 of 41 of patients or 49% of patients (21). Of note, the results obtained in our retrospective study in terms of pathologic complete response rate are lower than expected in both cohorts, although we cannot provide an explanation for this finding. Importantly, not only did we not identify any differences in cancer-specific survival or time to recurrence among patients treated with 3 vs. 4 cycles, but we reported that non-cancer specific survival was worse in patients receiving an additional cycle(HR= 2.53; 95 CI= 1.05- 6.10; p=0.046). When we analyzed all available baseline variables between the two cohorts, we did not identify any difference that may explain this finding. Considering that long-term platinum-induced cardiovascular toxicity has been shown in young patients treated for germ-cell tumors (22), we speculate that an additional cycle may increase mid- and long-term cardiovascular toxicity with an increased risk of death in a population who is generally at high or very cardiovascular high risk (males, elderly, heavy smokers). As reported by others (13), we confirmed that pathologic downstaging and complete response were associated with prognosis, with only 5 reported deaths among the 49 patients with complete response vs. 62 reported deaths among the 170 patients without complete response.

In conclusion, although we are well aware of the limitations of our retrospective study, which include the study design and the limited sample size, this study represents the largest specifically designed to capture potential differences in outcomes between 3 and 4 cycles of neo-adjuvant cisplatin-gemcitabine. We found that 3 vs. 4 cycles may be equally effective, with a signal of decreased overall mortality in patients who received less cycles. Our finding may be incorporated in novel combination prospective trials based on cisplatin+gemcitabine +immune checkpoint inhibitors. Prospective studies are warranted.

## Data Availability Statement

The raw data supporting the conclusions of this article will be made available by the authors, without undue reservation.

## Ethics Statement

Ethical review and approval was not required for the study on human participants in accordance with the local legislation and institutional requirements. Written informed consent for participation was not required for this study in accordance with the national legislation and the institutional requirements.

## Author Contributions

MF and CB contributed to the conception of the work and final version approval. All authors contributed to the article and approved the submitted version.

## Conflict of Interest

The authors declare that the research was conducted in the absence of any commercial or financial relationships that could be construed as a potential conflict of interest.
